# Neuroprotective Effect of Epalrestat on Hydrogen Peroxide-Induced Neurodegeneration in SH-SY5Y Cellular Model

**DOI:** 10.4014/jmb.2101.01002

**Published:** 2021-04-06

**Authors:** Sivakumar Lingappa, Muthugounder Subramanian Shivakumar, Thamilarasan Manivasagam, Somasundaram Thirugnanasambandan Somasundaram, Palaniappan Seedevi

**Affiliations:** 1 Centre of Advanced Study in Marine Biology, Faculty of Marine Sciences, Annamalai University, Parangipettai 608502, Tamilnadu, India; 2 Molecular Entomology Lab, Department of Biotechnology, Periyar University, Salem 636011, Tamilnadu, India; 3 Department of Biochemistry & Biotechnology, Faculty of Science, Annamalai University, Annamalainagar 608002, Tamilnadu, India; 4 Department of Environmental Science, Periyar University, Salem 636011, Tamilnadu, India

**Keywords:** Epalrestat, hydrogen peroxide, neuroprotection, oxidative stress, Tau pathology, SH-SY5Y cell line

## Abstract

Epalrestat (EPS) is a brain penetrant aldose reductase inhibitor, an approved drug currently used for the treatment of diabetic neuropathy. At near-plasma concentration, EPS induces glutathione biosynthesis, which in turn reduces oxidative stress in the neuronal cells. In this study, we found that EPS reduces neurodegeneration by inhibiting reactive oxygen species (ROS)-induced oxidative injury, mitochondrial membrane damage, apoptosis and tauopathy. EPS treatment up to 50 μM did not show any toxic effect on SH-SY5Y cell line (neuroblastoma cells). However, we observed toxic effect at a concentration of 100 μM and above. At 50 μM concentration, EPS showed better antioxidant activity against H_2_O_2_ (100 μM)-induced cytotoxicity, ROS formation and mitochondrial membrane damage in retinoic acid-differentiated SH-SY5Y cell line. Furthermore, our study revealed that 50 μM of EPS concentration reduced the glycogen synthase kinase-3 β (GSK3-β) expression and total tau protein level in H_2_O_2_ (100 μM)-treated cells. Findings from this study confirms the therapeutic efficacy of EPS on regulating Alzheimer's disease (AD) by regulating GSK3-β and total tau proteins phosphorylation, which helped to restore the cellular viability. This process could also reduce toxic fibrillary tangle formation and disease progression of AD. Therefore, it is our view that an optimal concentration of EPS therapy could decrease AD pathology by reducing tau phosphorylation through regulating the expression level of GSK3-β.

## Introduction

Alzheimer’s disease (AD) is one of the most common chronic neurodegenerative diseases and is associated with two types of pathological hallmarks in the brain. These biomarkers are extracellular plaque deposits mainly composed by amyloid-beta (Aβ) peptides and intracellular neurofibrillary tangles (NFT) formed by tau hyperphosphorylation [1]. Aβ peptides are deposited through enzymatic cleavage of amyloid precursor protein (APP) by β and γ-secretase enzymes [2]. In 2017, the Alzheimer’s Association surveyed the AD population worldwide and found 48 million people with early symptoms of the disease. The prevalence of the disease is expected to double by 2050. The most common symptoms are short-term memory loss, difficulty in finding words, trouble with visual-spatial understanding, reasoning, judgment and insight.

Reactive oxygen species (ROS) are the major key molecules in neurodegenerative diseases. Hydrogen peroxide (H_2_O_2_) exposure in cultured neuronal cells would cause an imbalance of ROS production and scavenging activities. This process could be widely used to study this oxidative stress inducer in cell line models [3]. H_2_O_2_-induced oxidative stress can cause hyperphosphorylation in tau protein and induce formation of toxic neurofibrillary tangles in the brain cells [4]. In addition, all-*trans*-retinoic acid (RA) induction would enhance the neuronal morphological differentiation of SH-SY5Y cells and augment Alzheimer's disease markers such as tau and GSK3-β expression [5]. Hence, H_2_O_2_-induced pathogenicity and RA differentiation in SH-SY5Y cells could serve as a suitable model system for studying neuronal cell degeneration.

Neurodegeneration is mainly correlated with mitochondrial dysfunction, ROS formation, activation of apoptotic pathways and elevation of iron and nitric oxide, which modify the homeostasis of antioxidant/oxidant levels in brain cells [6, 7]. These cellular mechanisms induce the Aβ plaque deposition, toxic NFT formation and thus cause neuronal degeneration [8].

Epalrestat (EPS) is a carboxylic acid derivative that inhibits aldose reductase, a rate-limiting enzyme of the polyol pathway. This drug is currently being used for the treatment of diabetic neuropathy [9]. Moreover, EPS also alleviates the high glucose-induced oxidative stress, mitochondrial membrane potential and inhibition of intracellular apoptotic pathway activation in cardiomyocytes model [10]. The therapeutic dose of EPS might be useful for improving oxidative stress-related diverse diseases including neurodegenerative diseases like Alzheimer's. In brain cells, EPS was also found to induce glutathione (GSH) biosynthesis resulting in enhanced resistance to oxidative stress [9]. Oxidative stress and low GSH level were said to be the key contributors of aging and chronic neurodegenerative diseases. Moreover, a recent study has found that EPS could enhance the synthesis of super oxide dismutase, catalase, and heme-oxygenase-1 (HO^-1^) [11].

ROS and the reduced glutathione are the major factors involved in the neurodegenerative pathogenesis of diseases such as AD. Recently, it has been demonstrated that diabetes may influence the rate of cognitive decline among patients with mild AD [12]. Diabetes is also recognized as a risk factor for the development of cognitive impairment and accelerated progression of AD [13]. Since EPS has various biological and therapeutic beneficial activities, this study aimed to evaluate the effect of EPS on ROS homeostasis and early apoptotic control in H_2_O_2_-induced oxidative stress in a neuronal cell line. In addition, EPS was also examined for its regulation on AD markers like GSK3-β and total tau. This study was conducted with SH-SY5Y cells in order to better understand EPS regulation in controlling oxidative stress-induced neurodegeneration and related disease progression.

## Materials and Methods

### Chemicals

Epalrestat, 3-(4,5- dimethylthiazol-2-yl)-2,5-diphenyltetrazolium bromide (MTT), 2',7'-diacetyl dichlorofluorescein (DCFHDA), Rhodamine 123 (Rh-123), and fetal bovine serum (FBS) were purchased from Sigma Aldrich, USA. Dulbecco’s modified Eagle’s medium (DMEM), ethylene diamine tetra acetic acid (EDTA), trypsin, Folin’s Ciocalteu reagent (FCR), trichloro acetic acid (TCA), and 4', 6-diamidino-2-phenylindole (DAPI) were purchased from Himedia, India. Anti-GSK3-β, anti-total tau, anti-β-actin and anti-rabbit secondary antibodies were purchased from Cell Signaling, USA.

### Cell Culture

SH-SY5Y neuroblastoma cells were obtained from the National Centre for Cell Science (NCCS), Pune, India. Cells were grown in DMEM/F12 Hams (1:1) medium supplemented with 10% v/v fetal bovine serum (FBS). Cells were maintained at 37°C under 5% CO_2_ (Eppendorf galaxy 170R, Germany) and 95% humidity. The medium was changed every two days for continued propagation of the cells.

### Neuronal Differentiation Induction

SH-SY5Y cells were grown in neuronal induction medium (NIM), which consisted of minimum essential medium (MEM) containing 5% FBS supplemented with 10 μM retinoic acid (RA). SH-SY5Y cells were grown in NIM for 6 days, and switched to serum-free NIM prior to treatment and harvesting RA-SH-SY5Y on day 7 [14]. The cellular morphology was documented using a TS-100 with NIS-E v3.1 (Nikon, Japan).

### MTT Assay

Cell viability was analyzed using MTT reduction assay [15]. Retinoic acid-treated RA-SH-SY5Y cells were seeded in 96-multiwell plates at 1 × 10^3^ cells/well and the cells were incubated at 37°C for 24 h. The cells were treated with increasing concentration of EPS (5, 10, 25, 50, 100, 200 μM) to optimize the LD_50_ value. Before treatment with EPS, the cells were preincubated with 100 μM H_2_O_2_ for 2 h. After 24 h incubation, cells were incubated with MTT (0.5 mg/ml) at 37°C for 4 h. The medium solution was removed and the cells were suspended in 200 μl of DMSO. The absorbance of formazan reduction was measured at 570 nm in a microplate reader.

### Measurement of Intracellular ROS Formation

The intensity of intracellular peroxide was quantified by treating the cells with dichloro-dihydro-fluorescein diacetate (DCFH-DA) [16]. The esterified fluorescent probe penetrated into the intracellular matrix of the cells and reacted with ROS to form fluorescent dichlorofluorescein (DCF). In brief, Control and EPS-treated RA-SH-SY5Y cells (3 × 10^3^ cells/ml) were incubated with 100 μl DCFH-DA (10 μM) for 30 min at 37°C. Cells were rinsed twice with phosphate-buffered saline (PBS) and observed under a fluorescent microscope (450-490 nm; blue filter). Fluorescence intensity was estimated with excitation and emission filters at 485 ± 10 nm and 530 ± 12.5 nm respectively using a spectrofluorometer (Shimadzu, RF-5301 PC). The results were represented with the fluorescence intensity in percentage variance (TS-100 with NIS-E v3.1, Nikon).

### Measurement of Mitochondrial Membrane Potential (ΔΨm)

Mitochondrial membrane potential (MMP) was determined fluorometrically using fluorescent rhodamine (Rh-123) dye, which resulted in photoluminescent quenching from polarized mitochondria [17]. The cells treated with EPS and H_2_O_2_ for 24 h were further incubated with 1 μg/ml of Rhodamine 123 for 15 min. Then the cells were rinsed with PBS, and observed under fluorescence microscopy at 450–490 nm. Fluorescent intensity was measured at 535 nm using a spectrofluorometer and the images were documented (TS-100 with NIS-E v3.1, Nikon).

### Assessment of Apoptosis

Fluorescent probe permeable dye acridine orange (AO) and non-permeable dye ethidium bromide (EtBr) were used to analyze RA-SH-SY5Y cell apoptosis by observing morphological assessment of nuclear bodies [18]. The four experimental groups were conducted with RA-SH-SY5Y cell line (3 × 10^3^ cells/ml). The first control group was maintained without any treatment. The second group was treated with H_2_O_2_ and the third group was treated with EPS and H_2_O_2_. The fourth group was maintained with EPS alone. The culture was incubated at 37°C with 5%CO_2_ for 24 h. Then, the treated cells were washed with PBS twice and stained with 1 μl of AO/EtBr (stock solution 100 μg/ml). These cells were incubated for 20 min at room temperature and washed with warm PBS to remove excess dye. Cellular morphology was observed using a fluorescent microscope (λEx/λEm = 490 nm/530 nm) and documented (TS-100 with NIS-E v3.1, Nikon). Fluorescent intensity was also measured at 535 nm using a spectrofluorometer.

### Nuclei Staining in Apoptotic Cells Using DAPI Stain

DAPI forms a fluorescent intercalative complex preferentially by attaching in the minor groove of AT-rich DNA sequences in damaged cells [19]. After treatment, the cells were washed with cold PBS thrice and fixed with paraformaldehyde (4%) in PBS solution for 10 min. The processed cells were washed again with PBS and treated with 0.25% Triton X-100 in PBS to permeabilize the cells for 10 min. After washing with PBS solution, the cells were stained with DAPI (5 μg/ml) for 10 min at RT. The stained cells were observed under a fluorescence microscope (TS-100 with NIS-E v3.1, Nikon) to confirm the presence of intercalative complex of DAPI in apoptotic cells exhibiting size-reduced nuclei, intense fluorescence and nuclear fragmentation.

### Western Blot Analysis

Western blot analysis was performed to study the EPS regulatory effect of GSK3-β and total tau protein expression in oxidative stress-induced RA-SH-SY5Y cells. After 24 h of incubation, the cells were lysed in 100 ml of ice-cold radio-immunoprecipitation assay (RIPA) buffer followed by centrifugation (10,000 ×*g*) at 4°C for 10 min to remove insoluble material. The protein content was quantified using Bradford’s method [20] and electrophoresed in 10% SDS-polyacrylamide gel then transferred on PVDF membrane by semi-dry transfer method. After blocking the membrane with 5% non-fat dry milk in tris-buffered saline, blots were incubated with primary antibodies GSK3β (1:1000), total tau (1:1000) and β-actin (1:1000) for overnight at 4°C. After washing excess unbound primary antibodies, the membrane was incubated with anti-rabbit HRP conjugated secondary antibody (1:2000), and the bands were detected by chemiluminescence staining using an ECL detection kit. The protein expression was quantified using an ImageJ densitometric analyser.

### Statistical Analysis

All experimental data were expressed as mean ± SD. The statistical significance was evaluated by one-way analysis of variance (ANOVA) using SPSS version 11.5 (SPSS, USA) and the individual comparison was analyzed by using Duncan’s Multiple Range Test (DMRT). A value of *p* < 0.05 was considered as significance.

## Results

### Retinoic Acid-Induced SH-SY5Y Cell Neurodifferentiation

**Fig. 1:**
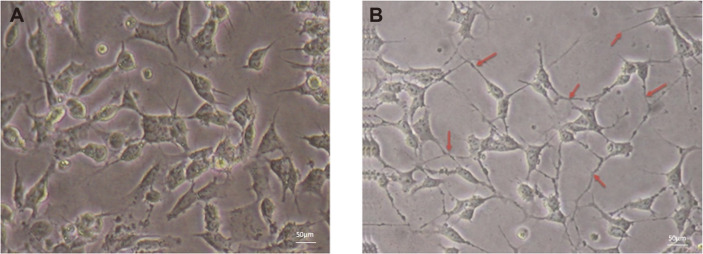
Neuronal differentiation. RA-induced neuronal differentiation and morphology of SH-SY5Y cells. (**A**) Undifferentiated SH-SY5Y cells with short or without neurites. (**B**) Red arrows show 10 μM RA-induced neurite outgrowth signifies neuronal differentiation of SH-SY5Y cells.

SH-SY5Y neuroblastoma cells treated with 10 μM of retinoic acid (RA) exhibited more elongated neurite growth than that of the control cells (Fig. 1). The morphological observation of the SH-SY5Y cells showed multiple neurite outgrowths along with increased axon span in most of the cells. In the differentiated SH-SY5Y cell population, more than 95% of the cells displayed neurite connections between their neighboring ones. On the other hand, only 10% of the untreated control cells showed such phenotypes. Hence, the differentiated cells were considered as an in vitro model to analyze the neuroprotective effect of EPS for further experiments.

### Effect of EPS on H_2_O_2_-Induced Cytotoxicity in RA-SH-SY5Y Cells

**Fig. 2:**
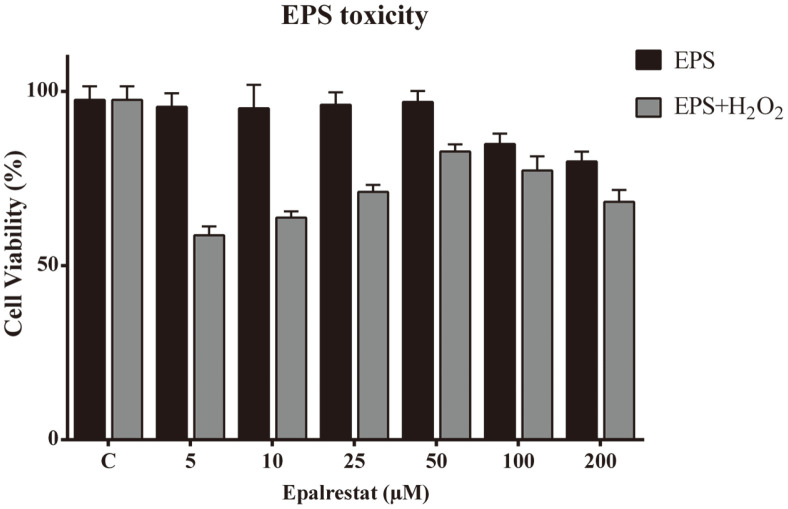
Epalrestat effect on H_2_O_2_ cellular encounter. The effect of epalrestat on 100 μM H_2_O_2_-induced cytotoxicity in SH-SY5Y neuroblastoma cells. All experimental inducer groups were pre-treated with 100 μM H_2_O_2_ and supplemented EPS (0-200 μM) for 24 h. All data are presented as the mean ± SD of triplicate independent experiments. *p < 0.05 vs control.

The effect of EPS was assessed on retinoic acid (10 μM)-differentiated SH-SY5Y cells by MTT assay. The level of formazan product formation in this assay is directly proportional to the number of viable cells. Fig. 2 represents the percentage of viable cells after their exposure to EPS at an increasing dosage concentration of different experimental groups (5, 10, 25, 50, 100, and 200 μM). The result showed that there were no conspicuous changes between control and 50 μM EPS-treated groups of cells. On the contrary, 100 and 200 μM (dosage concentration of EPS)-treated cells showed cytotoxicity (15-20%). At the same time, 5-50 μM EPS concentration was found to inhibit the H_2_O_2_ induced cytotoxicity and maintained cellular viability. But, at 100 μM and above concentration, the viability of the cells was found to decrease up to 30% in oxidative stress-induced cells (Fig. 2). Therefore, 50 μM EPS dosage was found to be the most suitable concentration for further investigation to evaluate its neuroprotective effect.

### EPS on H_2_O_2_-Induced Intracellular ROS Formation in Neuronal Cells

**Fig. 3:**
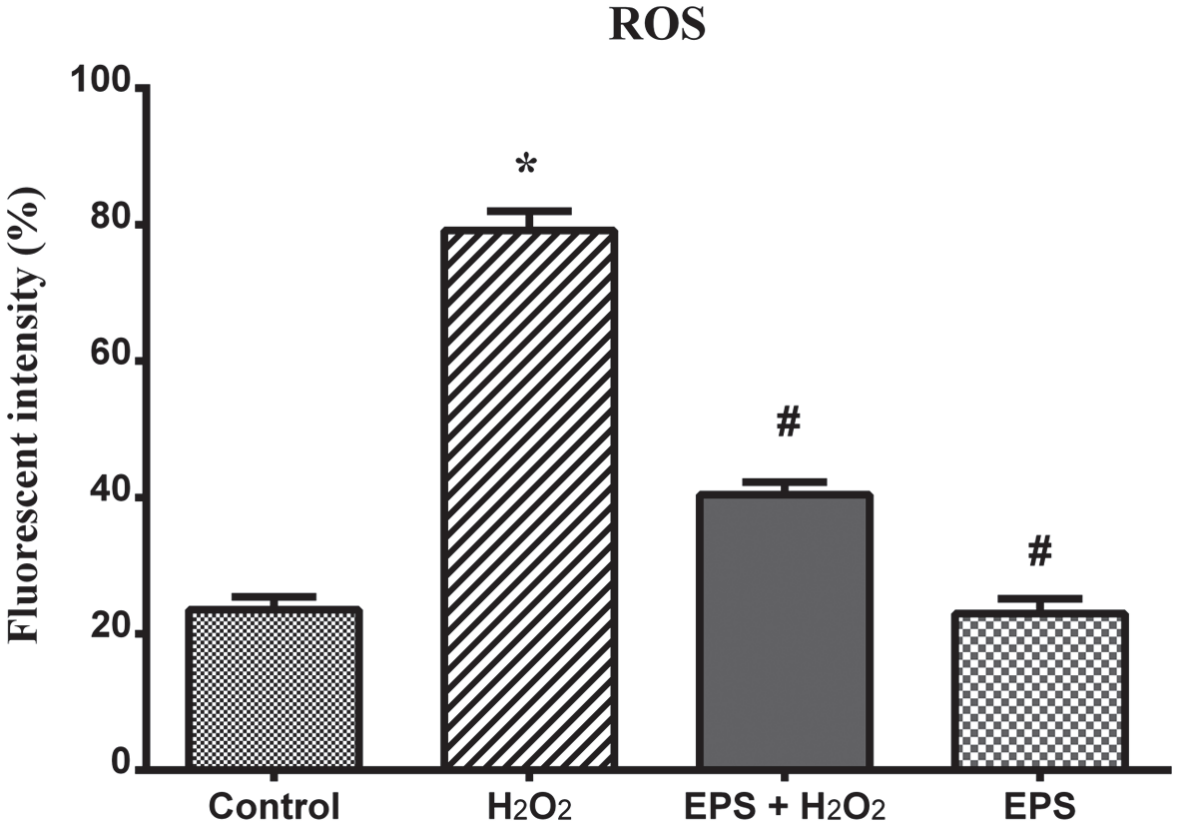
Effect of Epalrestat on inhibition of intracellular ROS formation by DCFH-DA staining. Graphical representation of fluorescent intensity shows that H_2_O_2_ (100 μM) treatment significantly increased the fluorescent intensity compared to control cells. Epalrestat (50 μM) treatment inhibited a significant level of ROS generation in H_2_O_2_-treated SHSY5Y cells. The cells treated with EPS alone remained as healthy as control cells. The fluorescent intensity was calculated as mean ± S.D. in each group. *p < 0.05 compared to control and #*p* < 0.05 compared to peroxide alone-treated group.

ROS formation inhibition activity of EPS was examined in RA-SH-SY5Y cell lines. Differentiated SH-SY5Y cells were treated with 100 μM H_2_O_2_ for 2 h and then the cells were exposed to 50 μM EPS. After 24 h of EPS treatment, the level of intracellular ROS concentration was measured using DCFH-DA fluorescence. The intensity of fluorescence is directly proportional to intracellular ROS concentration. Fig. 3 shows the enhanced ROS level corresponding to the increased green fluorescence intensity compared to control cells. In contrast, in EPS-treated (50 μM) cells, the intracellular ROS level decreased significantly by twofold. Therefore, EPS concentration was found to be optimal to inhibit ROS formation, subsequently reducing the ROS-induced neuronal damage and accordingly protecting the cells from deterioration.

### Mitochondrial Membrane Potential Effect of Epalrestat in SH-SY5Y Cells

**Fig. 4:**
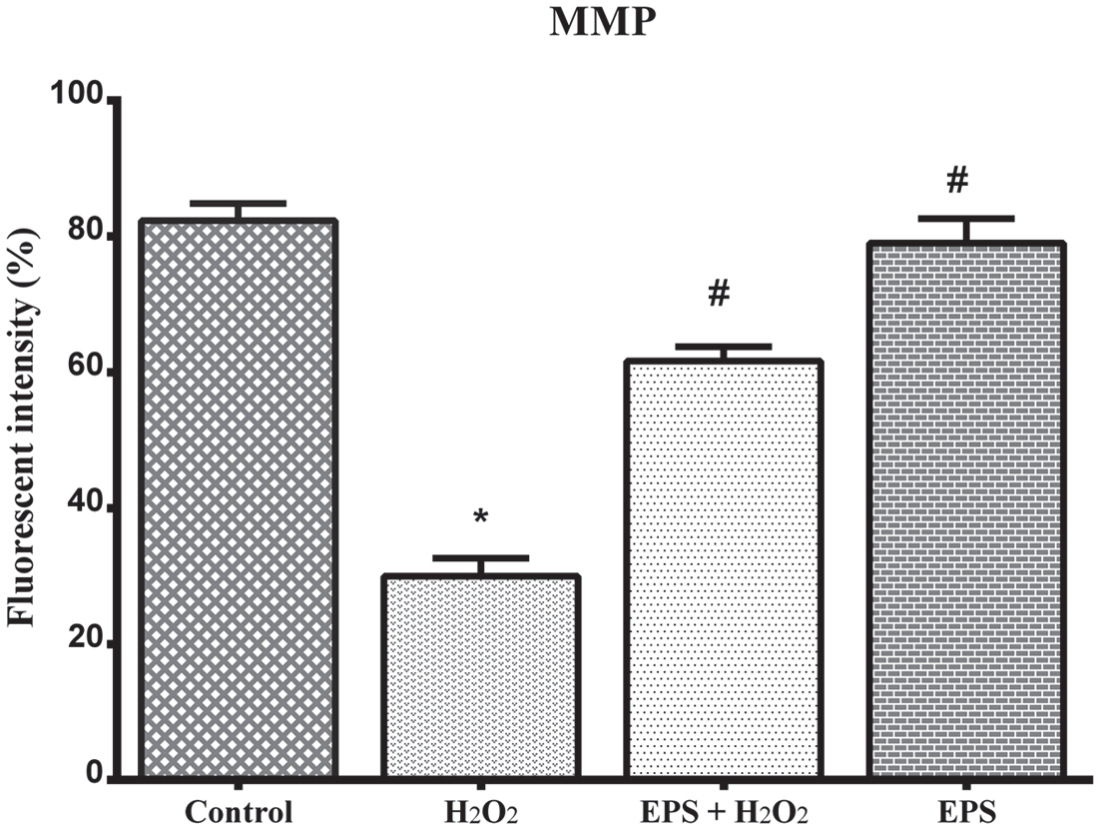
Epalrestat-reinforced ability of mitochondrion membrane by Rhodamine-123 staining. Graphical representation clearly displays the preventive effect of Epalrestat (50 μM) against H_2_O_2_ oxidative stress-induced mitochondrion membrane damage in RA-differentiated SH-SY5Y cells. H_2_O_2_ (100 μM) damaged the mitochondrial membrane more than 60% compared with control cells. In contrast, Epalrestat (50 μM) pre-treatment has protected significant level of MMP in H_2_O_2_- treated SH-SY5Y cells. The data are given as mean ± SD in each group. *p < 0.05 compared to control, #*p* < 0.05 compared to H_2_O_2_-treated group.

The mitochondrial membrane potential (MMP) was investigated by Rhodamine-123 staining. The positively charged lipophilic Rh-123 stain can directly pass through the cell membrane and exhibit green fluorescence by staining the mitochondrion. The H_2_O_2_-treated cells showed poor fluorescence intensity (30%) compared to untreated control cells (Fig. 4). Nonetheless, the EPS-treated cells showed increased green fluorescence intensity (64%) than H_2_O_2_ pre-treated inducer control cells. Enhancement of fluorescence intensity was reflected by the effect of EPS through mitochondrial membrane-bound fluorescent probe Rh-123 (64 ± 0.9%) in H_2_O_2_ pre-treated cells. Consequently, the result revealed the effect of EPS in inhibiting ROS formation and mitochondrion membrane protection.

### Epalrestat Influence on Oxidative Stress-Induced Cellular Damage

**Fig. 5:**
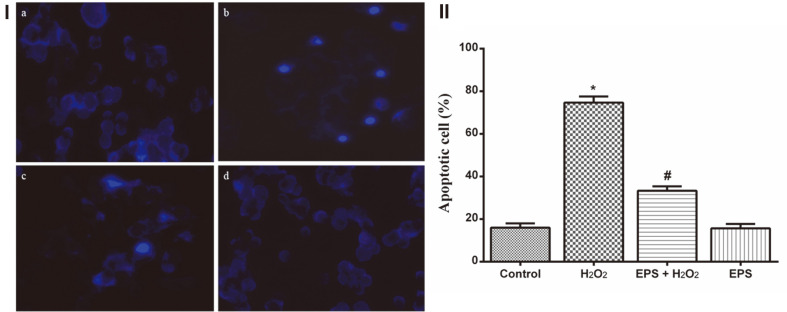
Apoptosis and DNA fragmentation inhibition effect of Epalrestat in RA-SH-SY5Y cells. (**I**) Photomicrograph showing the fluoresced DNA fragments stained by DAPI on peroxide-induced apoptosis caused in RA-SHSY5Y cells. **a**. Control, **b**. H_2_O_2_, **c**. Epalrestat + H_2_O_2_, and **d**. 50 μM Epalrestat treated. H_2_O_2_ (100 μM)-treated cells showing high number of fluoresced fragmented nuclei than Epalrestat (50 μM) treatment. (**II**) Graphical representation showing the morphological changes caused by apoptotic cell damage and the inhibition ability of Epalrestat on H_2_O_2_-treated RA-SH-SY5Y cells. H_2_O_2_ (100 μM) exposure has provoked programmed cell death but Epalrestat (50 μM) treatment rescued the neuronal cells from oxidative cellular damage. The data are given as mean ± SD in each group. *p < 0.05 compared to control, ^#^*p* < 0.05 compared to H_2_O_2_-treated group.

The dual staining with acridine orange/ethidium bromide (AO/EB) revealed the distinct characteristic of apoptotic cellular morphology and DNA damage in RA-SH-SY5Y cells. This experiment helped to distinguish the viable and late apoptotic cells. The viable cells exhibited uniform bright green nuclei and the late apoptotic cells had highly fragmented chromatins (Fig. 5II). SH-SY5Y cells treated with 100 μM hydrogen peroxide exhibited an orange-red fluorescence apoptotic body formation (73 ± 1.5%), while the necrotic cells showed orange to red nuclei. EPS-exposed, hydrogen peroxide-pre-treated cells were not found to have such significant number of orange-red apoptotic bodies, and instead displayed green-colored cellular morphology. Thus, this study revealed the effect of EPS in inhibiting peroxide stress-induced apoptotic cellular damage and also was found to protect the cells from oxidative stress-induced neurodegeneration.

### DNA Damage Restoration by EPS

The DNA fragmentation fortification effect of EPS was evaluated by DAPI staining in RA-SH-SY5Y cells. The nonviable cells were identified by observing the uniform bright blue-colored nuclei (damaged DNA fragments) and the viable cells did not show fluoresced nuclei. In this analysis, H_2_O_2_ treated cells showed bright blue luminescent nuclei, which revealed the fragmentation of DNA and indicated the formation of apoptotic bodies (Fig. 5IB). Like the control cells (Fig. 5IA), EPS-treated H_2_O_2_-exposed cells did not show any deformities and the cells were found to have intact nuclei (Fig. 5IC).

### Epalrestat Effect on GSK3-β and Total Tau Regulation

**Fig. 6:**
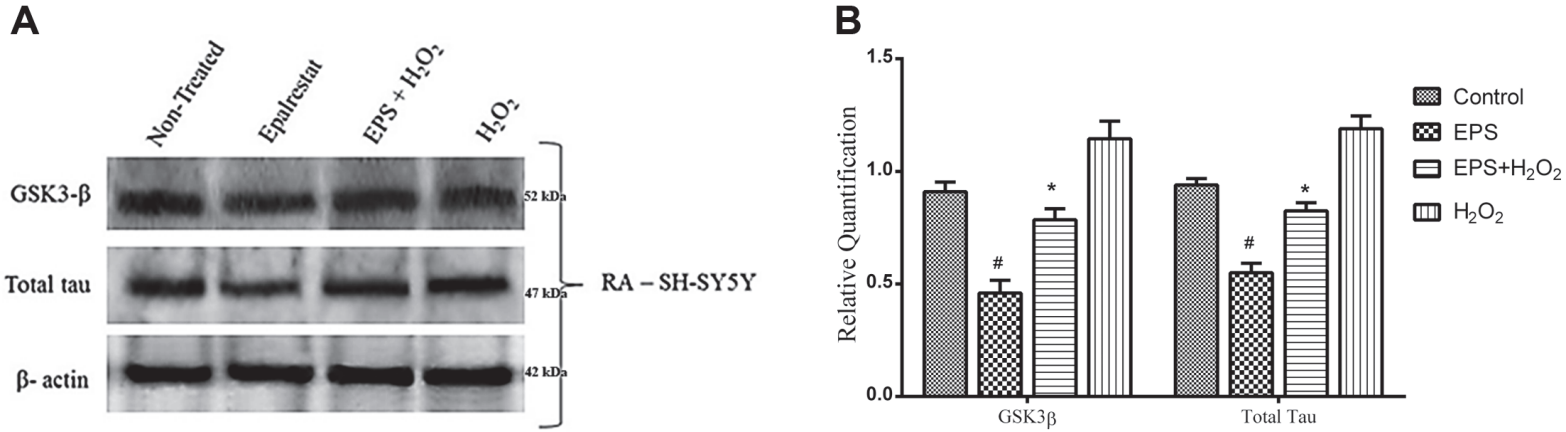
Effect of Epalrestat on GSK3-β and total tau expression in 100 μM peroxide-induced oxidative stress in RA-differentiated SH-SY5Y cells. (**A**) Western blot result. (**B**) Graphical representation of western blot results. 50 μM EPS treatment significantly decreased GSK3-β and t-tau expression in peroxide-supplemented and RA-differentiated neuronal cells. The densitometric values are expressed as arbitrary units and given as mean ± SD. *p < 0.05 compared to the peroxideinduced group and #*p* < 0.05 compared to un-treated control group.

The protein expression was analyzed for Alzheimer’s markers like GSK3-β and total tau (T-tau) in RA-differentiated SH-SY5Y cells by western blotting. It is well known that increased GSK3-β expression would enhance tau hyperphosphorylation. This condition could induce toxic intracellular neurofibrillary tangle formation and deposition, which would result in cellular degeneration. In this study, RA was used to enhance AD-type protein markers expression in SH-SY5Y cells. Subsequently, EPS treatment showed downregulation of GSK3-β protein (30%) in H_2_O_2_ stress-induced cells. Accordingly, it also reduced the level of total tau protein expression in SH-SY5Y cells (Fig. 6). Although there was a negligible variation in the expression of both GSK3-β and total tau protein between the control and EPS treated cells, there was no significant (*p* > 0.05) difference. Thus, it was concluded that 50 μM EPS treatment may prevent oxidative stress-induced cellular damage by regulating tau and GSK3-β protein expression.

## Discussion

EPS is a commercially available and approved drug to treat human diabetic neuropathy. It has been found that the neuronal tissue could easily absorb EPS and is metabolically involved in different types of signaling pathways [21]. Jaiswal *et al*. reported that the antioxidant activity of EPS could control the hyperphosphorylation and misregulation of tau protein and nerve cell damage in the hippocampus region [22]. Hyperphosphorylation of tau causes NFT formation, which is a major intracellular sign that Alzheimer’s disease is affecting the hippocampus and cortex region of the brain [1]. The hippocampus region is specifically important for learning and memory and is the first area to be affected in AD. The present study was focused on investigating the neuroprotective effect of EPS by analyzing the effect of EPS on AD markers.

Retinoic acid (RA)-differentiated SH-SY5Y cells enhanced the neuron-specific protein expression for the regulation of their structural and functional mechanism [23, 24]. The increased retinol concentration could enhance tau phosphorylation, β-amyloid peptide formation, reactive species production and immunocontent of α-synuclein secretion [5, 25]. Therefore, RA-differentiated SH-SY5Y cells were found to be the most suitable model system to assess the neuroprotective effect of EPS.

Yama *et al*. [25] reported that 50 μM EPS dramatically reduced hydrogen peroxide-induced cytotoxicity in bovine aortic endothelial cells and increased the GSH biosynthesis by activating Nrf2. Consistently, this study also provides evidence of the cytotoxic protection of EPS by inhibiting 67% of H_2_O_2_-induced cellular damage in RA-differentiated SH-SY5Y cells. However, EPS concentration greater than 100 μM reduces this cytoprotective effect. In addition, this EPS concentration was found to enhance the expression of caspase-3 and subsequently induce apoptotic process in neuronal cells [9]. Additionally, we found that EPS concentration greater than 100 μM may reduce the fortification effect and upregulate apoptotic markers in endothelial cells [11, 25]. Anne *et al*. [14] suggested that the therapeutic dose of EPS might be a new tactic to treat various oxidative stress-induced diseases like Alzheimer’s, Parkinson’s and arthrosclerosis. Concordantly, this study revealed that 50μM concentration of EPS can effectively inhibit H_2_O_2_-induced cellular damage.

In ROS-stimulated intrinsic apoptosis, the mitochondrial membrane permeabilization increases by forming pores in the outer mitochondrial membrane [26]. The release of inner mitochondrial proteins into the cytosol triggers the pro-apoptotic cascade and in turn cell death. An increase in mitochondrion membrane transition pore size is a sign of increased free radical formation, mitochondrial membrane distension and loss of mitochondrion membrane potential [27]. The mitochondrial membrane permeabilization is a main stimulus of necrotic and apoptotic cell death [28]. Evidenced by the lack of mitochondrial membrane damaged cells to up take rhodamine staining, it appears that EPS treatment reduces ROS generation and protects the cell from mitochondrial membrane transition pore formation. Recent reports also suggest that EPS can control oxidative stress by regulating intracellular GSH synthesis through the instigation of Nrf2 pathway [25, 29] resulting in the protection of neuronal cells.

ROS stimulate anomalous regulations in neuronal ion channels and induces cellular (sensory and motor) damage. K^+^ ion channel oxidation might be a major cause for neurodegenerative diseases like Alzheimer’s and Parkinson’s [30]. Increased ROS generation would increase the extracellular β-amyloid deposition and induce the cleavage of tau protein which is the major cause of AD pathology [31]. According to our findings, EPS would overcome these ROS-induced affects by stalling caspase activation, tau protein cleavage and subsequent NFT formation. Effectively, this would result in the inhibition of mitochondrion membrane polarization and apoptosis.

During the course of hydrogen peroxide treatment, the neuronal cell membrane became permeable to ethidium bromide (EB) [32]. Normally, EB enters the cytosol and stains the damaged DNA fragments an orange-red color. However, EPS treatment rescued the H_2_O_2_-stressed neuronal cells by reducing the cell membrane permeability. In addition, DAPI staining also revealed the effect of EPS in protecting the neuronal cells from DNA damage.

The abnormal regulation of GSK3 protein, a multifunctional kinase, might induce neurodegeneration by enhancing the expression of apoptotic markers and necrotic proteins like β-catenin and Bax [33]. This study showed that hydrogen peroxide supplement can augment GSK3-β and total tau protein expression in RA-differentiated SH-SY5Y cells. Therefore, GSK3-β misregulation would increase total tau protein through hyperphosphorylation and lead to the onset of Alzheimer disease [34]. Nevertheless, EPS supplement was found to significantly reduce the level of GSK3-β and total tau expression in oxidative stress-induced neuronal cells. In addition, Jaiswal *et al*. [22] reported that 57 mg/kg EPS has an ability to inhibit total tau protein expression in the hippocampus tissue in diabetic rat models.

In conclusion, our study provides evidence for the role of EPS in preventing cellular damage in the brain. EPS does this by obstructing oxidative stress, mitochondrial membrane damage, apoptotic cascade activation and through meticulous regulation of GSK3-β and tau protein. Therefore, EPS might be an effective therapeutic molecule to combat Alzheimer’s and other related neuropathology.
